# Genomic Expedition: Deciphering Human Adenovirus Strains from the 2023 Outbreak in West Bengal, India: Insights into Viral Evolution and Molecular Epidemiology

**DOI:** 10.3390/v16010159

**Published:** 2024-01-21

**Authors:** Ananya Chatterjee, Uttaran Bhattacharjee, Rudrak Gupta, Ashis Debnath, Agniva Majumdar, Ritubrita Saha, Mamta Chawla-Sarkar, Alok Kumar Chakrabarti, Shanta Dutta

**Affiliations:** 1 Virus Research and Diagnostic Laboratory, ICMR-National Institute of Cholera and Enteric Diseases, Kolkata 700010, West Bengal, India; ananya866@gmail.com (A.C.); rudrakgupta@gmail.com (R.G.); drashisdebnath.10@gmail.com (A.D.); agniva05@outlook.com (A.M.); drshantadutta@gmail.com (S.D.); 2 Division of Virology, ICMR-National Institute of Cholera and Enteric Diseases, Kolkata 700010, West Bengal, India; uttaran.inspire21@gmail.com (U.B.); saharitubrita@gmail.com (R.S.); chawlam70@gmail.com (M.C.-S.)

**Keywords:** respiratory adenovirus, outbreak, whole genome sequencing, recombinant strain, genetic characterisation

## Abstract

Understanding the genetic dynamics of circulating Human Adenovirus (HAdV) types is pivotal for effectively managing outbreaks and devising targeted interventions. During the West Bengal outbreak of 2022–2023, an investigation into the genetic characteristics and outbreak potential of circulating HAdV types was conducted. Twenty-four randomly selected samples underwent whole-genome sequencing. Analysis revealed a prevalent recombinant strain, merging type 3 and type 7 of human mastadenovirus B1 (HAd-B1) species, indicating the emergence of recent strains of species B in India. Furthermore, distinctions in VA-RNAs and the E3 region suggested that current circulating strains of human mastadenovirus B1 (HAd-B1) possess the capacity to evade host immunity, endure longer within hosts, and cause severe respiratory infections. This study underscores the significance of evaluating the complete genome sequence of HAdV isolates to glean insights into their outbreak potential and the severity of associated illnesses.

## 1. Introduction

Human adenovirus (HAdVs) belongs to the family Adenoviridae and Genus Mastadenovirus. Adenoviruses are non-enveloped, icosahedral, linear double-stranded DNA viruses with diameters of 80–110 nm and DNA genomes of approximately 25–48 kbp in length surrounded by a non-enveloped icosahedron capsid. The icosahedral capsid of adenoviruses consists of 240 capsomeres without a vertex (hexons) and 12 capsomeres with a vertex (pentons). The fiber knob is formed by protein IV homotrimers and has three structural domains: the tail, which is attached to the base of the penton, the axis of characteristic length, and the distal bulge [1]. The core is composed of DNA protected with inverted terminal repeats (ITR) of 145 bases in length. Four early genes (E1–E4) are transcribed for viral proteins before the replication of viral DNA and the five late genes (L1–L5) are transcribed for structural proteins after the viral DNA replication [2]. More than 110 types of HAdV have been reported so far [3]. Multiple types of HAdVs are grouped into species. Seven species (A–G) of the HAdVs are grouped based on their immune reaction [4]. The pathogenicity of many types is unexplored [1] HAdV infection creates mild respiratory conditions (such as pharyngitis) and conjunctivitis. Conjunctivitis due to HAdV infections is more common in Southeast Asia. HAdV replicates very well in epithelial cells in the primary barrier of the gastrointestinal tract, respiratory tracts, conjunctiva, and urinary bladder [1]. Severe acute respiratory infections, gastroenteritis with dehydration, hemorrhagic cystitis, and meningoencephalitis are the outcome of fatal HAdV infections, often observed in outbreak cases [5]. A fatal form of HAdV infection is mostly observed in both pediatric populations, especially those under 5 years of age, and immunocompromised patients with comorbidities. On average, 5% to 10% of all febrile illnesses in infants and preschool-aged patients are observed due to HAdV infections. Although viral infections are usually self-limiting, persistent infections are also found in the preschool population and immunosuppressed patients [6]. Infection of HAdV type5 is mostly observed in humans. About 71–73% seroprevalence due to HAdV4 or HAdV5 was reported in Washington D.C, USA [7] and China [8]. In addition, 73% seroprevalence was observed in pediatric patients due to HAdV5 infection [8]. HAdV species B is further subdivided into two sub-species, B1 and B2. HAdV3, HAdV7, HAdV16, HAdV21, and HAdV50 belong to sub-species B1 and HAdV11, HAdV14, HAdV34, and HAdV35 belong to sub-species B2. HAdV3, HAdV7, and HAdV21 are commonly known causative agents of severe respiratory infections which have caused epidemic outbreaks in the past and so they are very well studied. On the other hand, HAdV16 and the recently identified HAdV50 are rarely detected and so are not well studied. A large amount of genetic variability is also observed in the HAdV3, 7, and 21 types [9].

In both developed and developing countries, adenovirus infections and infrequent outbreaks are quite common throughout the year [10,11]. Since December 2022, there has been a noticeable increase in the number of childhood pneumonia cases in West Bengal, India. Adenovirus was identified in the samples as the causative agent related to illness [12]. Since it was associated with the severity of the disease, it is important to assess the entire genome sequence of HAdV isolates to conduct molecular analysis of adenovirus strains involved in the West Bengal outbreak. In this study, we have analyzed whole genome sequences of 24 randomly selected samples of the West Bengal outbreak from December 2022–March 2023 with the aim of determining the genetic composition of the circulating HAdV types, providing valuable insights into their potential for causing outbreaks and the severity of associated illnesses.

## 2. Materials and Methods

### 2.1. Sample Source

Nasopharyngeal and oropharyngeal swabs from patients with severe acute respiratory illness (SARI) admitted to different tertiary care hospitals in West Bengal and patients coming to the outdoor facility with influenza-like illness (ILI) were referred to the Regional Virus Research and Diagnostic Laboratory, ICMR-NICED, Kolkata for testing.

### 2.2. Real-Time PCR of Respiratory Viral Panel

RNA extractions from 200 μL nasopharyngeal/oropharyngeal swabs in viral transport media (VTM) were performed using the QIAamp^®^ viral RNA mini kit (Qiagen, Germany, Cat. No. 52906) according to the manufacturer’s instructions. The extracted RNA was screened for the presence of respiratory pathogen using a respiratory viral panel (InfA-H1N1, InfA-H3N2, Inf-B, RSV, hMPV, PIV, Adenovirus, Rhinovirus) with AgPath-ID™ One-Step RT-PCR Reagents (Cat No. 4387391). Thermal cycling was performed at 50 °C for 30 min for reverse transcription, followed by 95 °C for 5 min, then 45 cycles of 95 °C for 15 s, and 55 °C for 30 s for annealing and amplification. Data acquisition was performed at 55 °C.

### 2.3. Amplicon Preparation for Ion Torrent NGS Platform (Ion GeneStudio S5 System)

The whole genome of respiratory Adenovirus was amplified in five amplicons of ~7 Kbp size using self-designed primer sequences (Supporting Information Table S1). Invitrogen™ Platinum™ SuperFi™ DNA Polymerase (Catalog number: 12351050) was used to amplify the segments. Thermal cycling was performed at 95 °C for 5 min for one cycle, followed by 30 cycles of 95 °C for 15 s for denaturation, 53/57 °C (53 °C for fragments 2 and 3 and 57 °C for fragments 1, 4, and 5) for 30 s for annealing, and 72 °C for 3 min 30 s for extension. The amplified products were gel purified from 0.8% agarose gel using the QIAquick Gel Extraction Kit (Catalog number: 28704) as per the manufacturer’s instructions.

### 2.4. Library Preparation for the Ion Torrent NGS Platform (Ion GeneStudio S5 System)

The purified PCR fragments were further purified using AMPure XP magnetic beads (Catalog No.: A63881). The purified products were then quantified using Qubit 4 Fluorometers using Qubit™ 1X dsDNA High Sensitivity (HS) and Broad Range (BR) Assay Kits (Catalog number: Q33231). The concentration for all five amplicons for each sample was adjusted to the lowest amplicon concentration among the five and then pooled together for each sample. The 100 ng of pooled amplicons for each sample were sheared to ~450 bp using Ion Shear™ Plus Enzyme Mix II provided in the Ion Xpress™ Plus Fragment Library Kit (Catalog number: 4471269) as per the manufacturer’s instructions. The Adaptor and Barcode sequence provided in the Ion Xpress™ Barcode Adapters Kit (Catalog number: 4474517) was then ligated and nick-repaired to the sheared products using DNA ligase and the Nick repair enzyme provided in the Ion Xpress™ Plus Fragment Library Kit. The ~490–500 bp ligated products were then selected using E-Gel™ Size Select™ II Agarose Gel, 2% (Cat. No. G661012). The size-selected product was then amplified using Platinum™ PCR SuperMix High Fidelity provided in the Ion Xpress™ Plus Fragment Library Kit. The amplified library was purified using AMPure XP magnetic beads. The prepared library was then quantified using Qubit™ 1X dsDNA High Sensitivity (HS) and Broad Range (BR) Assay Kits. Prepared libraries for each sample were then diluted to a concentration of 100 pM and pooled. Afterwards, 40pM of the pooled library was loaded on the Ion Chef instrument for clonal amplification and chip loading was performed according to the manufacturer’s instructions. The loaded chip was then inserted into the Ion GeneStudio S5 for sequencing and initial analysis, using assembler SPAdes [13], coverage analysis, and variant calling plugins in the Ion Torrent Suite 5.18.1 software.

### 2.5. Sanger Sequencing of the 5′ ITR End and 3′ ITR End

The 5′ ITR regions and 3′ ITR end were amplified by self-designed primer sequence (Supporting Information Table S1) using Invitrogen™ Platinum™ SuperFi™ DNA Polymerase (Catalog number: 12351050) as per the manufacturer’s protocol. Thermal cycling was performed at 95 °C for 5 min for initial denaturation, followed by 30 cycles of 95 °C for 15 s, 53 °C for 30 s for annealing, and 72 °C for 30 s for extension. The amplified products were gel purified from 0.8% agarose gel using the QIAquick Gel Extraction Kit (Catalog number: 28704) as per the manufacturer’s instructions. Sanger sequencing PCR was performed using the Applied Biosystems™ BigDye™ Terminator v3.1 Cycle Sequencing Kit (Catalog number: 4337455) as per the manufacturer’s protocol. PCR clean-up was achieved using the EDTA-Sodium acetate ethanol precipitation protocol, followed by a 70% ethanol wash. The final product was re-suspended in HiDi formamide and sequenced in the ABI 3700 genetic analyzer.

### 2.6. Bioinformatic Analysis

The reference-based consensus from each sample’s VCF files was generated using the “bcftools Consensus” algorithm in https://usegalaxy.org/ (accessed on 14 April 2023). The Sanger sequences were visualized, edited, and aligned, and the consensus was prepared using MEGA X version 10.2.2 [14] and BioEdit software version 7.2.5 to get the complete whole genome sequence of the 24 samples. The resulting FASTA sequences were aligned and inspected using MEGA X software. Phylogenetic trees were generated using MEGA X software, employing the maximum likelihood estimation with 1000 bootstrap replications. The tertiary and secondary RNA structures of the VA-I region were determined using the trRosettaRNA [15] and Forna (force-directed RNA) [16] web servers, respectively. The homology model of PKR protein was generated using the SWISS-MODEL server [17] and validated with PROCHECK [18]. The VA-I RNA and PKR complex structure was generated using the HDOCK server [19,20,21,22,23]. We investigated the potential recombination events in the Indian HAdVs’ genomes in this study. The potential recombinants, parental sequences, and the possible recombination breakpoint were detected using the RDP, Geneconv, Maximum Chi-Square, Chimaera, BootScan, and SisterScan methods as implemented in RDP4 [24]. In this study, an isolate was designated as recombinant when at least four methods of RDP4 detected it.

## 3. Results

### 3.1. Genetic Characterisation of Adenovirus-Positive Clinical Samples Using Whole Genome Sequence Analysis

Since December 2022, there has been a noticeable increase in the number of childhood pneumonia cases in West Bengal, India. In response to this situation, the Virus Research and Diagnostic Laboratory (VRDL) and Division of Virology of Indian Council of Medical Research, National Institute of Cholera and Enteric Diseases (ICMR-NICED), located in Kolkata, India have taken on the task of identifying the primary agents responsible for these pneumonia cases. Screening of the child pneumonia samples for the respiratory viral pathogen identified adenovirus infections. Whole genome sequencing was performed on HAdV-positive patient samples obtained from 24 randomly selected hospitalized patients during the West Bengal outbreak between December 2022 and March 2023. Samples that were taken randomly for this study were found to be from patients belonging to the age group of <5 years, with sixteen (66.67%) male and eight (33.33%) female patients. Among the 24 samples, 12 (50%) had a history of ICU admission, including seven (29.17%) deceased outcomes (comprising five (71.43%) male and two (28.57%) female patients see Supporting Information Table S2 for further clinical information). Among the deceased cases, the most common symptom was breathlessness (100%), followed by coughing (85.71%) and fever (85.71%). Other symptoms were nasal discharge/stuffiness (71.43%) and vomiting/nausea (14.29%). On examination, wheezing (85.71%) and crepitations (85.71%) were the most common signs, followed by lower chest in-drawing (42.86%), nasal flaring (28.57%), accessory muscles used in breathing (14.29%), and apnea (8.33%).

Whole genome homology searches using BLAST [25] for all 24 samples showed homology to the human mastadenovirus B1 species. Genome length varied between 35246–35361 and the GC % of all samples was ~51%. The BLAST search of the 24 whole genome sequences revealed that 21 samples showed similarity with type 7 while three samples were similar to type 3. On the other hand, hexon, penton, and fiber genetic resemblance indicated that, out of 24 samples, one sample (NICED/23-13/2909) belonged to type 3 and the remaining 23 samples were recombinant strains between type 3 and type 7 HAdVs (Table 1). Among the recombinant strains, 21 samples belonged to type 7, of which 20 samples exhibited H7F3P7 genetic constituent and one sample (NICED/23-19/3312) showed H3F3P7 genetic constituent. The remaining two samples (NICED/23-01/1914 and NICED/23-12/2908) belonged to type 3, displaying genetic constituent H7F3P7 and H3F3P7, respectively. All the deceased cases were analyzed as adenovirus type 7 with H7F3P7 genetic constitution except one (NICED/23-19/3312), which belonged to H3F3P7 (Table 1). In total, 24 adenovirus whole genome sequences generated by NGS were submitted to the NCBI gene bank, as described in Supporting Information Table S3.

### 3.2. Phylogenetic Analysis

**Figure 1:**
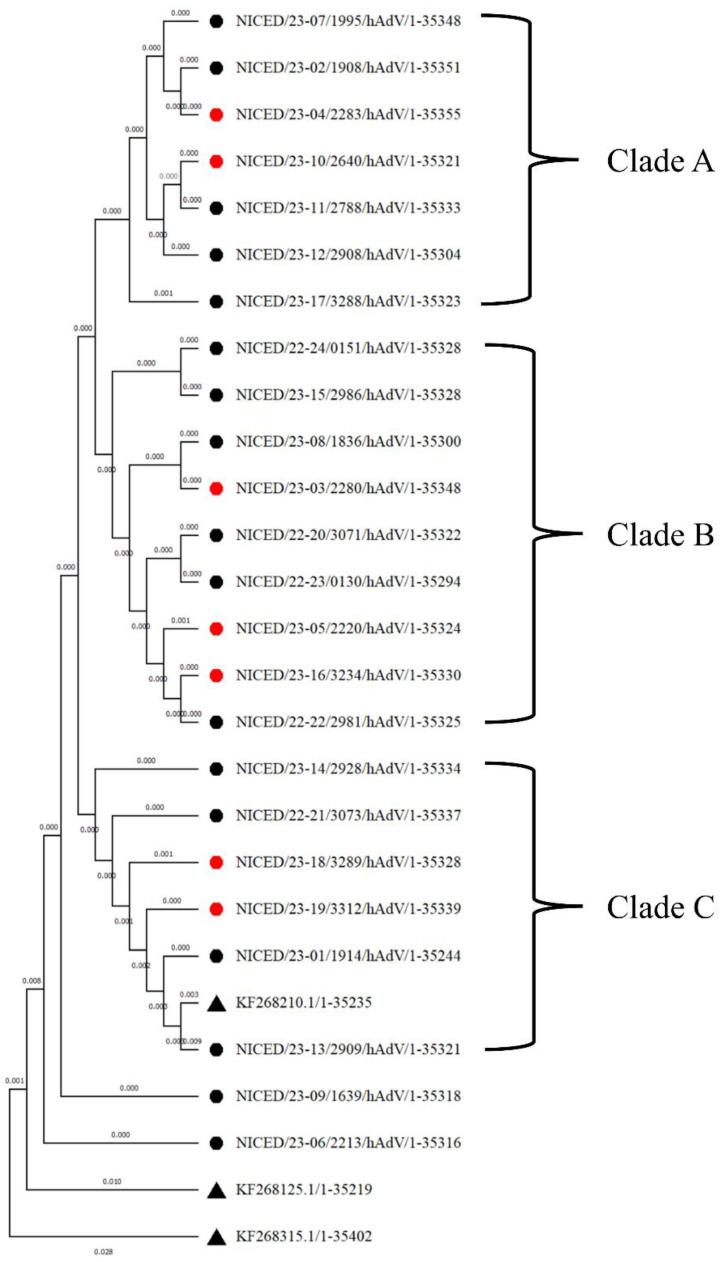
Phylogenetic analysis of 24 WGS samples. The samples marked with ▲ are reference sequences derived from NCBI, KF268315 (HAdV16), KF268125 (HAdV7), and KF268210 (HAdV3). ● Sequences of samples recovered from infection ● Sequences of samples which had fatal outcomes due to adenovirus infection. All samples are grouped into 3 clades with two outliers, NICED/23-06/2213 and NICED/23-09/1639.

Phylogenetic analysis of the whole-genome sequences of 24 samples reveals that NICED/23-06/2213 and NICED/23-09/1639 form two separate clades which are closely related to type 7. The rest of the 22 samples were clustered into three clades; clade A consists of seven samples (NICED/23-07/1995, NICED/23-02/1908, NICED/23-04/2283, NICED/23-10/2640, NICED/23-11/2788, NICED/23-12/2908, and NICED/23-17/3288), Clade B consists of nine samples (NICED/23-24/0151, NICED/23-15/2986, NICED/23-08/1836, NICED/23-03/2280, NICED/22-20/3071, NICED/23-23/0130, NICED/23-05/2220, NICED/23-16/3234, and NICED/22-22/2981), and clade C consists of six samples (NICED/23-14/2928, NICED/23-21/3073, NICED/23-18/3289, NICED/23-19/3312, NICED/23-01/1914, and NICED/23-13/2909). Among the seven samples of clade A, six samples belong to genotype 7[H7F3P7] and the remaining one, NICED/23-12/2908, belongs to genotype 3[H7F3P7]. All nine samples of clade B were detected as genotype 7[H7F3P7]. However, clade C consists of a variety of genotypes, with three samples belonging to genotype 7[H7F3P7], and the remaining three samples belonging to genotype 7[H3F3P7], 3[H3F3P7], and 3[H3F3P3], which were branched with a type 3 reference sequence (Figure 1).

**Figure 2:**
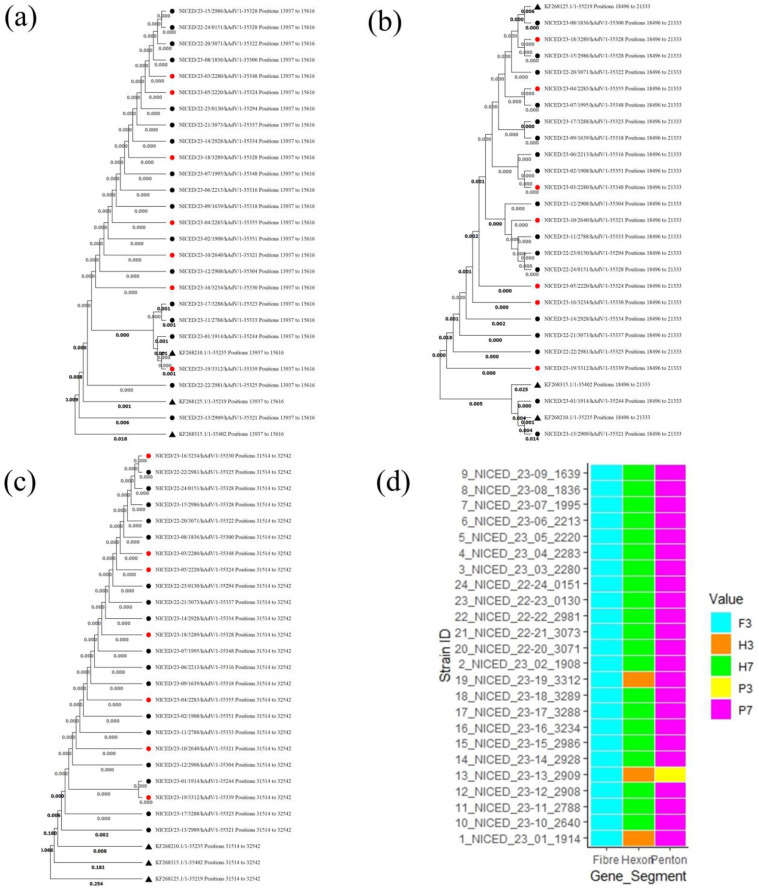
Phylogenetic analysis based on Penton, Hexon, and Fiber genes. The samples marked with ▲ are reference sequences derived from NCBI, KF268315 (HAdV16), KF268125 (HAdV7), andKF268210 (HAdV3). ● Represents sequences of samples recovered from infection ● Represents sequences of samples which had fatal outcomes due to adenovirus infection. (**a**) Phylogenetic tree based on the Penton gene, (**b**) Phylogenetic tree based on the Hexon gene, (**c**) Phylogenetic tree based on the Fiber gene, and (**d**) Pictorial representation of genotypes based on Fiber, Hexon, and Penton genes.

Phylogenetic analysis based on Penton, Hexon, and Fiber regions shows a different clustering pattern. In Hexon region-based phylogenetic analysis, two major clades were formed, a clade A cluster with 22 samples with a type 7 reference sequence and a Clade B cluster with two samples (NICED/23-01/1914 and NICED/23-13/2909) with type 3 and type 16 reference sequences (Figure 2b). Fiber region-based phylogenetic analysis showed a single-clade clustering, showing a close relation to type 3 reference sequences (Figure 2c). The Penton region-based phylogenetic tree was also clustered in a single clade with a sub-clade consisting of NICED/23-17/3288, NICED/23-11/2788, NICED/23-01/1914, NICED/23-19/3312, and a type 3 reference sequence (Figure 2a).

### 3.3. Inspection of Multiple Sequence Alignment (MSA)

**Figure 3:**
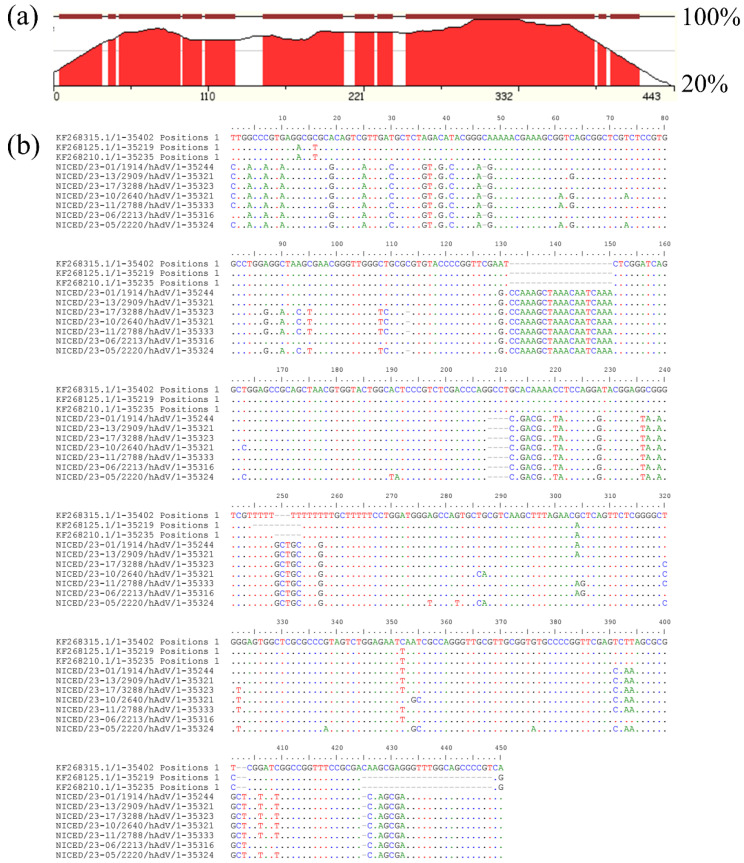
(**a**) zPicture (https://zpicture.dcode.org/) accessed on 17 May 2023 analysis based on the BlastZ local alignment algorithm for pairwise comparison between reference sequence KF268125 and NICED/23-17/3288 and (**b**) Overall variation observed in the VA-I and VA-II regions in sequences samples.

**Figure 4:**
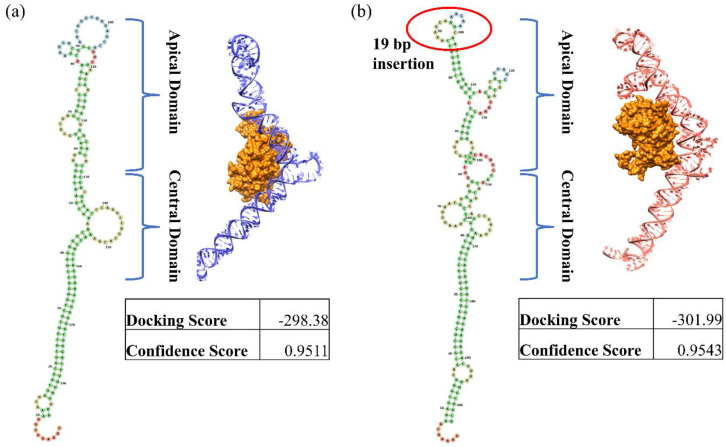
Comparison of change in secondary and tertiary VA-I RNA structures and their PKR binding. (**a**) Representative structure of type 3 and type 7 VA-I RNA (KF268210) (**b**) Representative structure of new VA-I RNA variant from WGS-sequenced samples (NICED/23-01/1914).

On close inspection, the multiple sequence alignment (MSA) of the whole genome shows many variations within the region 10358–10807, which codes for virus-associated RNA genes (VA-I and VA-II) (Figure 3). Apart from multiple point mutations, nineteen nucleotide insertions (10489–10507) and four nucleotide deletions (10565–10568) were present in the VA region. Secondary structure determination showed a structural change in the apical and central domains of the VAI region, where the 19 bp insertion forms an additional stem-loop structure in the apical region (Figure 4) and a more complex central domain. Protein kinase R (PKR) is known to interact with double-stranded RNAs of 10 to 15 base pairs in length. Additionally, PKR engages with intricately structured single-stranded RNAs that feature bulges, loops, pseudoknots, and single-stranded tails. Structural studies have shown that for stronger interactions, both apical and central domains are involved. The docking study of the VAI RNA and PKR shows that the new variant shows a stronger binding compared to the previously known variant. A miRBase database search of the VA-I region of sequenced samples shows homology with miR 197-3p, which was not observed in type 3 and 7 VA-I regions.

**Figure 5:**
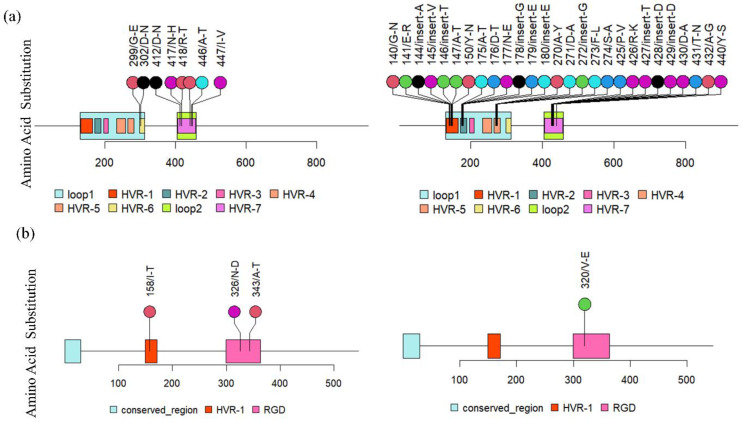
The lollipop plot illustrates amino acid (AA) substitutions (**a**) The immunogenic domain of the hexon protein in Human Adenovirus type 3 and type 7. Illustrates the AA substitutions in the hypervariable regions (HVR) within loop 1 (HVR 1–6) and loop 2 (HVR 7) of the hexon protein in the NICED/23-13/2909 strain of type 3 human adenovirus. (**b**) Amino acid (AA) substitutions in the conserved region, hypervariable region 1 (HVR 1), Arg–Gly–Asp (RGD) domain on the Penton base protein in human adenovirus type 3 and type 7. Illustrates the AA substitutions in the Penton base protein of the NICED/23-13/2909 strain which belongs to HAdV type 3.

The hexon gene was found to be a hybrid of the HAdV3 [H3F3P7] and HAdV7 [H7F7P7] genotype reference sequences. Among the two 9 bp signature regions, 18926–18934 (AA145 VTT) and 19017–19025 (AA 174 TTE), of adenovirus type 3, the first signature region was present in all the 24 sequences. However, the second signature sequence was found only in four sequences, i.e., NICED/23-14/2928, NICED/23-16/3234, NICED/23-21/3073, and NICED/22-22/2981. These variations were observed in the variable region 3 (V3) of the hexon gene (Figure 5). Compared with the reference strain of human adenovirus type 3 (GenBank KF268210), the NICED/23-13/2909 strains had two amino acid (AA) substitutions (299/G-E and 302/D-N) in the loop 1 region, and six AA substitutions (412/D-N, 417/N-H, 418/R-T, 441/T-P, 446/A-T, and 447/I-V) in the loop 2 region of the hexon protein. In context, compared with the reference strain of human adenovirus type 7 (GenBank KF268125), the strains belonging to this type in this study had eleven AA substitutions (140/G-N, 141/E-R, 147/A-T, 150/Y-N, 175/A-T, 176/D-T, 177/N-E, 270/A-Y, 271/D-A, 273/F-L, and 274/S-A) and seven insertions (141/insert-A, 145/insert-V, 146/insert-T, 178/insert-G, 179/insert-E, 180/insert-E, and 272/insert-G) in loop 1 as well as six AA substitutions (425/P-V, 426/R-K, 430/D-A, 431/T-N, 432/A-G, and 440/Y-S), and three insertions (427/insert-T, 428/insert-D, and 429/insert-D) in loop 2 of the hexon gene.

**Figure 6:**
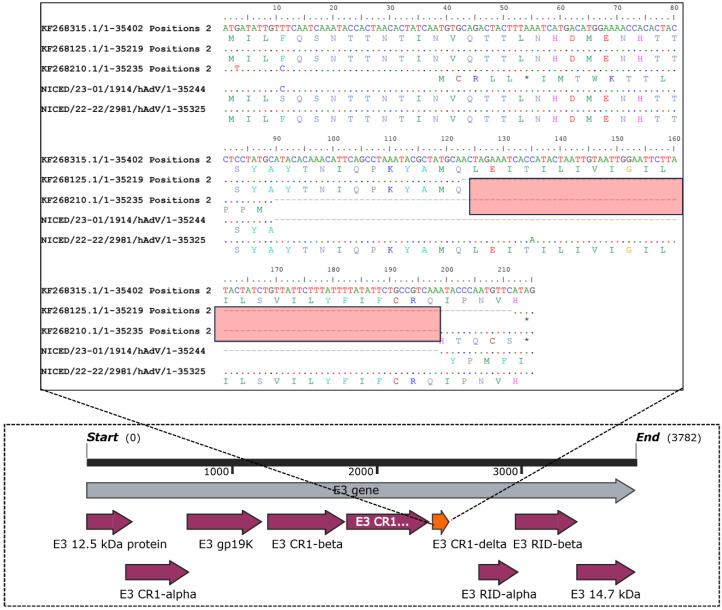
Schematic representation of the whole E3 gene (marked in grey), different E3 products (marked in magenta) and the insertion observed in the E3 CR1-delta region (marked in orange).

Moreover, the AA substitution at position 150/Y-N was identified in eight strains (NICED/23-10/2640, NICED/23-11/2788, NICED/23-12/2908, NICED/23-14/2928, NICED/22-21/3073, NICED/22-22/2981, NICED/23-23/0130, and NICED/23-24/0151), while substitutions at positions 175/A-T and 176/D-T were found in three strains each (NICED/23-16/3234, NICED/22-21/3073, and NICED/22-22/2981), and substitutions at position 177/N-E were observed in four strains (NICED/23-14/2928, NICED/23-16/3234, NICED/22-21/3073, and NICED/22-22/2981). In contrast, insertions at positions 178, 179, and 180 were exclusively detected in four strains (NICED/23-14/2928, NICED/23-16/3234, NICED/22-21/3073, and NICED/22-22/2981) within our study. All other mentioned AA substitutions and insertions related to type 7 were present in all strains of type 7 HAdv in this investigation. Regarding AA substitutions in the penton base, only the NICED/23-13/2909 strain exhibited one substitution (158/I-T) in the hypervariable region 1 (HVR-1) and two substitutions (326/N-D and 343/A-T) in the arginine-glycine-aspartic acid (RGD) region of the penton base. The hexon assembly protein shows 12 bp deletions 25895–25906 (AA 741 EGAA) in all sequences except NICED/23-01/1914 and NICED/23-13/3312. A 76 bp insertion was present in all the sequenced samples except NICED/23-01/1914 in the E3-RD1 Delta gene at the 3′ end, leading to a ~7.8 kDa protein instead of 3.3 kDa and 4.5 kDa proteins in types 3 and 7, respectively. A homology search of the E3 region of all the samples except NICED/23-01/1914 showed homology with a type 16 reference sequence (Figure 6).

### 3.4. Recombinant Analysis

**Figure 7:**
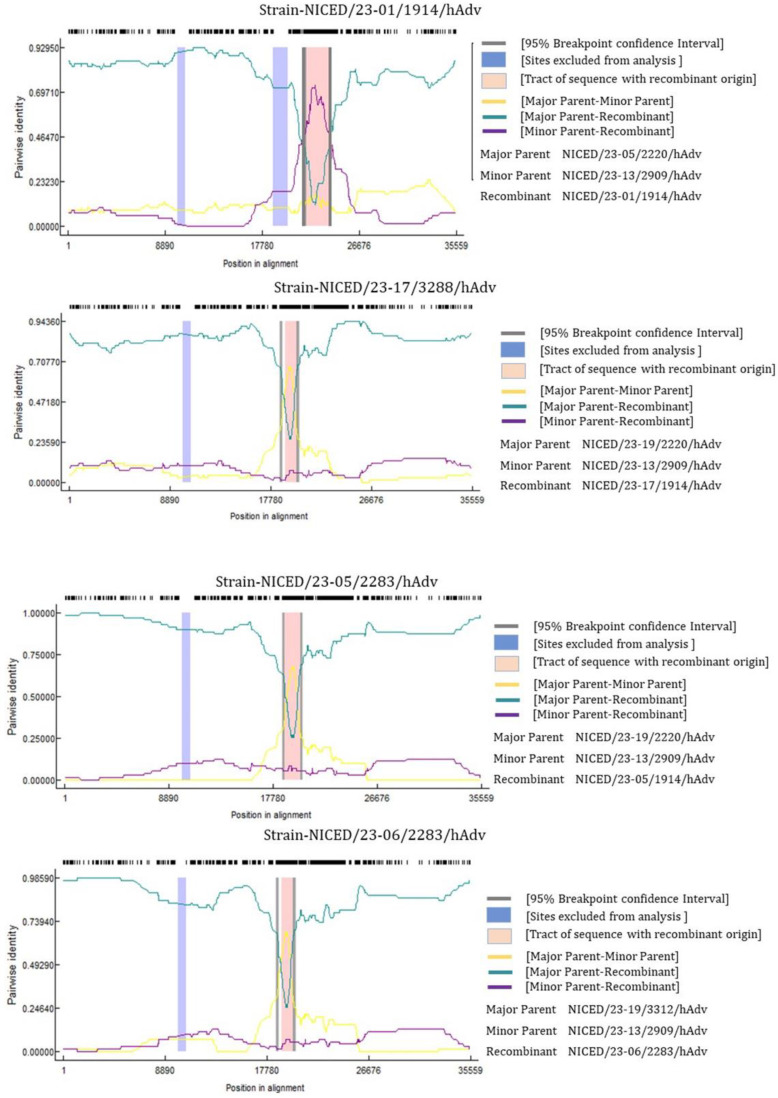
Recombination analysis using RDP 4. The figure displays the major recombinant sequences identified in this study: NICED/23-01/1914, NICED/23-05/2220, NICED/23-06/2283, and NICED/23-17/3288. Additionally, the potential major and minor parents of these recombinant strains are depicted, along with the sequence break points.

The recombination study provided valuable insights into the genetic makeup of various viral strains. Among the analyzed strains, NICED/23-01/1914, NICED/23-05/2220, NICED/23-06/2283, and NICED/23-17/3288 were identified as the major recombinant sequences. Of particular interest, NICED/23-13/2909, NICED/23-19/3312, and NICED/23-05/2220 were identified as potential parent strains involved in the recombination event (Figure 7). This finding suggests that the recombinant strain likely emerged from the genetic mixing of recent circulating strains belonging to species B of India. Notably, the presence of NICED/23-05/2220 as the major parental strain in the recombination process of NICED/23-01/1914 is a significant observation. This indicates that this particular strain may have played a crucial role in driving the adaptation and spread of the virus. In context, the phylogenetic study based on Fiber, Hexon, and Penton gene segments of adenovirus shows that the recombinant strains NICED/23-01/1914 and NICED/23-17/3288 clustered with an identical fiber-hexon-penton (H3F3P7) combination to their possible parental strains NICED/23-05/2220, NICED/23-13/2909, and NICED/23-19/3312. Interestingly, the NICED/23-06/2283 recombinant strain of this study clustered with the H7F3P3 genotype in the phylogenetic tree, and the Hexon- and Penton-based phylogeny represents a divergent cluster from its possible parental strains, NICED/23-19/3312 (H3F3P7) and NICED/23-13/2909 (H3F3P7). In the recombination analysis, maximum breakpoint densities occurred near the Hexon and Penton regions. The amino acid substitutions in the recombinant strain represent prior amino acid substitution patterns that might improve the survival of the recombinant strain, preventing the formation of novel chimeric proteins and maintaining the existing protein fold [26].

## 4. Discussion

The scarcity of reported HAdV strains in India is attributed to the limited number of studies conducted on HAdV epidemiology and molecular evolution in the country. Our study has contributed towards the enrichment of the HAdV whole genome sequence database from India, furthering our understanding of circulating strains. The categorization of adenoviruses depends on the major capsid genes such as the Penton, Hexon, and Fiber genes, which have proven to be effective in understanding the epidemiology of circulating HAdV strains and provided valued insights into the genetic diversity as well as the interrelationships among the strains. Furthermore, homologous recombination of HAdV capsid genes significantly influences the progression of recombinant HAdVs and can influence HAdV pathogenicity. Therefore, molecular characterization and phylogenetic studies of HAdV exclusively depending on single gene sequences may not provide adequate molecular resolution. Hence, a whole genome sequencing approach helps to explore the phylogenomic relationships of the current circulating strains of adenoviruses [27,28]. This study reflected the details analysis of the whole genome sequences of 24 randomly selected samples from the West Bengal outbreak from December 2022–March 2023.

Through comparative analysis of the whole genome sequences obtained from the 24 samples collected during the West Bengal outbreak, it was observed that genetic recombination, spontaneous mutation, and gene shuffling occurred between the circulating strains of human mastadenovirus B1 serotypes. The adenovirus genome is known to undergo significant variation due to nucleotide insertions, deletions, substitutions, and recombination. As evident from previous observations, intra-serotype variability is quite prominent in adenoviruses, particularly in types HAdV 7, HAdV 3, and HAdV 21 [9,29]. Among the 24 sequences analyzed in our study, 21 samples showed homology with type 7, and three samples displayed homology with type 3. With regard to clinical outcomes, genotype 7[H7F3P7] showed more virulence. However, their genetic composition varied considerably based on the Penton, Hexon, and Fiber region sequences.

By conducting a comparative analysis of the adenovirus Hexon gene, it was possible to identify four conserved regions and three variable regions [30]. These variable regions are distributed among the conserved regions and denoted as C1-V1-C2-V2-C3-V3-C4 [29,30]. The variability observed in the Hexon gene in our study is present in variable region 3 (V3). The variability evident within the Hexon gene was further supported by our in silico recombination study, which recognized a maximum breakpoint near the Hexon region. The AA substitutions within the hypervariable regions 1–7 (loops 1 and 2) of the Hexon gene played an important role in developing vaccines and drugs against HAdV [31,32]. The RGD loop interacts with αvβ3 or αvβ5 integrins to facilitate the endocytosis process of the virus and HVR1 may be a target of neutralizing antibodies [33]. Therefore, analyzing the amino acid substitutions within the interactive domains of Hexon and Penton bases emerges as a valuable avenue for potential interventions. The identified substitutions and insertions may have implications for the virus’s virulence, host interactions, and potential immune responses, warranting further investigation. Genetic variability was also evident in the adenovirus-associated RNAs, as well as in the VA-I and VA-II regions. VA-I and VA-II are non-coding structured RNAs that are abundantly expressed during the late phase of the infection. These virus-associated RNAs are known to sabotage host innate immune systems and inhibit protein kinase R (PKR), which is involved in interferon response activation [34,35]. The homology of the VA-I region of all sequenced samples showed similarity with miR-197. miR-197 is known to downregulate IL-18 [36], and the downregulation of IL-18 in turn downregulates INF-γ which is known to regulate both innate and adaptive antiviral immunity [37].

Adenovirus E3 genes vary largely in different species as six to nine E3 proteins are expressed in a species-dependent manner. The E3 genes are known to modulate the cell-mediated immune response and apoptosis of infected cells. The E3 CR1-Delta proteins are one of the known conserved regions (CR) and are known to play a complex role in modulating host immune responses. However, the actual mechanism needs to be deciphered further [38,39,40]. Therefore, the insertion of 25 amino acids (showing similarity with type 16, a newly discovered adenovirus type) in the N-terminal region of E3 CR1-Delta indicates an active and functional E3 CR1-Delta protein and possible modulation of the host immune response. Furthermore, variation in both VA-RNAs and the E3 region suggests that the current circulating strains of human mastadenovirus B1 have a great ability to escape the host immune system, persist longer in the host, and create severe respiratory infections as observed in the hospitalized pediatric population in West Bengal.

The previous finding highlights a significant aspect of the emergence of novel HAdV pathogens and suggests recombination events occurring between different genotypes within the same species [41,42]. This recombination process creates a strong genetic link between the newly formed viral variants due to shared sequences of high homology. The recombination results of this study present intriguing insights into the genetic makeup of various strains of the virus through a recombination analysis and suggest that recent circulating strains in West Bengal, India belonging to genotype B played a crucial role in the emergence of the recombinant strains. Phylogenetic study and genetic recombination analysis have shed light on the dynamic nature of the virus’s genetic composition, the role of recombination in its evolution, and the significance of specific strains in driving its adaptation and transmission. Taken together, genetic recombination, base insertion, deletion, and spontaneous mutations all suggest the emergence of a new variant of human mastadenovirus B which is highly likely to cause epidemics and is capable of increasing the severity of the adenovirus infection. Furthermore, the presence of multiple circulating recombinant strains of HAdV and disease severity warrants a claim for regular surveillance and genetic characterization of the circulating adenovirus species.

## 5. Conclusions

The exploration into the strains of human mastadenovirus B1 during the West Bengal outbreak from December 2022 to March 2023 unraveled a rich tapestry of genetic complexities and recombination episodes. Our study illuminated the complexities within adenovirus genomes, emphasizing the role of recombination, mutation, and gene shuffling in driving the emergence of novel variants. Our study revealed distinct genetic variations in the VA, Hexon, and E3 region sequences of adenoviral genomes. The variability observed in the VA and E3 CR1-Delta regions of the adenoviral genome was particularly noteworthy, suggesting an active role in modulating the host’s immune response which may have potentially induced severe respiratory infections, as evidenced in the hospitalized pediatric population in West Bengal. This understanding is pivotal in devising effective strategies for the containment, treatment, and prevention of the evolving landscape of adenovirus variants.

## Figures and Tables

**Table 1:** Genetic characterization of whole genome-sequenced samples with clinical details and outcomes. The homology match based on WGS and the genotype of each sample as determined by Penton, Hexon, and Fiber homology searches.

Sl.	Patient	WG Homology	Genotype Based on, Hexon Fiber, Penton Genes	Co-Infection	ICU	Oxygen	Final
No	ID				Admission	Requirement	Outcome
1	1914	3 (KF268210.1, 99.21%)	3[H3F3P7]	No	No	No	Recovered
2	1908	7 (KF268125.1, 99.25%)	7[H7F3P7]	No	Yes	Yes	Recovered
3	2280	7 (KF268125.1, 99.21%)	7[H7F3P7]	PIV	Yes	Yes	Death
4	2283	7 (KF268125.1, 99.25%)	7[H7F3P7]	No	Yes	Yes	Death
5	2220	7 (KF268125.1, 99.14%)	7[H7F3P7]	No	Yes	Yes	Death
6	2213	7 (KF268125.1, 99.28%)	7[H7F3P7]	Rhino	No	No	Recovered
7	1995	7 (KF268125.1, 99.26%)	7[H7F3P7]	No	No	No	Recovered
8	1836	7 (KF268125.1, 99.16%)	7[H7F3P7]	No	No	Yes	Recovered
9	1639	7 (KF268125.1, 99.24%)	7[H7F3P7]	No	Not Available	Not Available	Not Available
10	2640	7 (KF268125.1, 99.19%)	7[H7F3P7]	No	Yes	Yes	Death
11	2788	7 (KF268125.1, 99.23%)	7[H7F3P7]	No	Not Available	Not Available	Not Available
12	2908	3 (KF268132, 97.75%)	3[H7F3P7]	No	Yes	Yes	Recovered
13	2909	3 (AY599834.1, 99.35%)	3[H3F3P3]	No	Yes	Yes	Recovered
14	2928	7 (KF268125.1, 99.19%)	7[H7F3P7]	No	Not Available	Not Available	Not Available
15	2986	7 (KF268125.1, 99.22%)	7[H7F3P7]	PIV	Yes	Yes	Recovered
16	3234	7 (KF268125.1, 99.16%)	7[H7F3P7]	No	Yes	Yes	Death
17	3288	7 (KF268125.1, 99.17%)	7[H7F3P7]	No	No	No	Recovered
18	3289	7 (KF268125.1, 99.10%)	7[H7F3P7]	RSV	Yes	Yes	Death
19	3312	7 (KF268125.1, 98.84%)	7[H3F3P7]	No	Yes	Yes	Death
20	3071	7 (KF268125.1, 99.21%)	7[H7F3P7]	No	No	No	Recovered
21	3073	7 (KF268125.1, 99.18%)	7[H7F3P7]	No	Not Available	Not Available	Not Available
22	2981	7 (KF268125.1, 99.14%)	7[H7F3P7]	No	No	No	Recovered
23	130	7 (KF268125.1, 99.05%)	7[H7F3P7]	No	Yes	Yes	Recovered
24	151	7 (KF268125.1, 99.21%)	7[H7F3P7]	No	No	No	Recovered

## Data Availability

The data that support the findings of this study are available in GenBank at https://www.ncbi.nlm.nih.gov/genbank/ under the Accession number OR039269, OR089143-OR089150, OR130164-OR130178. The clinical data not mentioned already in the manuscript is available from the corresponding author upon reasonable request.

## Supplementary Materials

The following supporting information can be downloaded at: https://www.mdpi.com/article/10.3390/v16010159/s1. Supporting Information Table S1: Primers for amplicon preparation for NGS and Sanger sequencing. Primers 1–10 were used for amplicon preparation for NGS and 11–16 were used for Sanger sequencing for end filling. Supporting Information Table S2: Clinical symptoms and signs of 24 WGS samples. Supporting Information Table S3: GenBank accession Number of sequences submitted in NCBI.
