# Supplementary material for: Genomic Expedition: Deciphering Human Adenovirus Strains from the 2023 Outbreak in West Bengal, India: Insights into Viral Evolution and Molecular Epidemiology
Source: Viruses. 2024 Jan 21;16(1):159. doi: 10.3390/v16010159 (PMC10820069; doi:10.3390/v16010159)
Supplement: Supplementary file 1 [file viruses-16-00159-s001.zip › viruses-2799132-supplementary.pdf]

Supporting Information Table S1: Primers for amplicon preparation for NGS and Sanger sequencing. Primers 1-10 were used for amplicon preparation for NGS and 11-16 were used for Sanger sequencing for end filling.

Sl. No	Sequence ID	Sequence (5'-3')	Location
1.	Ad_1F_Ion	ACGTGACTTATGTGGGAGGAGCT	136-155
2.	Ad_1R_Ion	TCTCGACTACTGTGCCCTGGAT	6895-6915
3.	Ad_2F_Ion	GCACAGTAGTCGAGAGTTTCCTGGA	6986-6926
4.	Ad_2R_Ion	GGCCTCAGCAAAGAGTCATTCAAG	13980-14003
5.	Ad_3F_Ion	AACTGGGGAGAATAACCCGC	13933-13953
6.	Ad_3R_Ion	TGAAGCGGTGTTGTGAGCCA	20927-20945
7.	Ad_4F_Ion	TCACCAGCATCAACCTCTATGCCA	20893-20917
8.	Ad_4R_Ion	CCAGCTGTACACCTGCGTTA	27867-27886
9.	Ad_5F_Ion	TGGTGTACCAGGAAACTCCCGCT	27794-27816
10.	Ad_6RR_Ion	CCCATTACGTCAGTTTAGGGCTTG	35358-35380
11.	ITR F	ATACCTTATAGATGGAATGGTGCC	5-29
12.	SAd_1R	AGCTCCGGACAGTCCAACTTA	1028-1048
13.	SAd_36F	ACA AGA CAA GCC ACA GGG TCT CCA	34940-34964
14.	SAd_36R	ACC TTA TAG ATG GAA TGG TGC C	35620-35641
15.	ITR R (Sanger Sequencing Primer)	TCACGTCATTTTCCCACGGC	118-138
16.	SAd_36FF (Sanger Sequencing Primer)	CCG AAA CTG CGT CAG CAG GGA AA	35421-35443

Supporting Information Table S2: Clinical symptoms and signs of 24 WGS samples.

Sl. No	Patient ID	Age	Sex	Genotype	ICU Admission	Final Outcome	Symptoms							
							Fever	Cough	Breathlessness	Nasal discharge/stuffiness	Sputum production	Vomiting/ nausea	Seizures	Earache/ discharge
1	1914	0Y 6M	Male	3[H3F3P7]	No	Recovered	YES	YES	YES	NO	NO	NO	NO	NO
2	1908	0Y 9M	Male	7[H7F3P7]	Yes	Recovered	YES	YES	YES	YES	NO	YES	YES	NO
3	2280	0Y 5M	Male	7[H7F3P7]	Yes	Death	YES	YES	YES	YES	NO	NO	NO	NO
4	2283	1Y	Male	7[H7F3P7]	Yes	Death	YES	NO	YES	YES	NO	NO	NO	NO
5	2220	0Y 9M	Male	7[H7F3P7]	Yes	Death	YES	YES	YES	YES	NO	YES	NO	NO
6	2213	0Y 5M	Male	7[H7F3P7]	No	Recovered	YES	YES	YES	NO	NO	NO	NO	NO
7	1995	1Y 4M	Female	7[H7F3P7]	No	Recovered	YES	YES	YES	YES	NO	NO	NO	NO
8	1836	0Y 10M	Male	7[H7F3P7]	No	Recovered	YES	YES	YES	YES	NO	NO	NO	NO
9	1639	0Y 6M	Male	7[H7F3P7]	Not Available	Not Available	YES	YES	YES	YES	NO	NO	NO	NO
10	2640	0Y 7M	Female	7[H7F3P7]	Yes	Death	YES	YES	YES	YES	NO	NO	NO	NO
11	2788	0Y 9M	Male	7[H7F3P7]	Not Available	Not Available	YES	YES	YES	NO	NO	NO	NO	NO
12	2908	1Y	Female	3[H7F3P7]	Yes	Recovered	YES	YES	NO	YES	NO	NO	NO	NO
13	2909	5Y	Male	3[H3F3P3]	Yes	Recovered	YES	YES	YES	YES	NO	NO	NO	NO
14	2928	0Y 7M	Male	7[H7F3P7]	Not Available	Not Available	YES	YES	NO	NO	YES	NO	YES	NO
15	2986	1Y	Male	7[H7F3P7]	Yes	Recovered	YES	YES	YES	YES	NO	YES	NO	NO
16	3234	1Y	Male	7[H7F3P7]	Yes	Death	YES	YES	YES	NO	NO	NO	NO	NO
17	3288	0Y 7M	Female	7[H7F3P7]	No	Recovered	YES	YES	YES	YES	NO	NO	NO	NO
18	3289	1Y	Male	7[H7F3P7]	Yes	Death	NO	YES	YES	YES	NOT KNOWN	NOT KNOWN	NO	NOT KNOWN
19	3312	0Y 11M	Female	7[H3F3P7]	Yes	Death	YES	YES	YES	NO	NO	NO	NO	NO
20	3071	0Y 11M	Female	7[H7F3P7]	No	Recovered	YES	YES	NO	YES	NO	NO	NO	NO
21	3073	0Y 8M	Female	7[H7F3P7]	Not Available	Not Available	NO	YES	NO	NO	NO	NO	NO	NO
22	2981	0Y 7M	Female	7[H7F3P7]	No	Recovered	YES	YES	NO	NO	NO	NO	NO	NO
23	130	0Y 11M	Male	7[H7F3P7]	Yes	Recovered	YES	YES	YES	YES	NO	NO	NO	NO
24	151	3Y 4M	Male	7[H7F3P7]	No	Recovered	YES	YES	NO	NO	NO	NO	NO	NO

Sl. No	Patient ID	Signs								
		Hemoptysis	Wheeze	Crepitations	Lower chest in-drawing	Nasal flaring	Accessory muscles used for breathing	Grunting	Stridor in calm patient	Apnea
1	1914	NO	YES	YES	YES	NO	NO	NO	NO	NO
2	1908	NO	YES	NO	YES	YES	YES	NO	NO	NO
3	2280	NO	YES	YES	YES	NO	NO	NO	NO	YES
4	2283	NO	YES	YES	NO	NO	NO	NO	NO	NO
5	2220	NO	YES	YES	NO	NO	NO	NO	NO	NO
6	2213	NO	YES	YES	YES	NO	NO	NO	NO	NO
7	1995	NO	YES	NO	NO	NO	NO	NO	NO	NO
8	1836	NO	YES	YES	NO	YES	NO	NO	NO	NO
9	1639	NO	YES	NO	YES	YES	YES	NO	NO	NO
10	2640	NO	YES	YES	YES	NO	NO	NO	NO	NO
11	2788	NO	YES	YES	YES	YES	YES	YES	NO	NO
12	2908	NO	YES	YES	NO	NO	NO	NO	NO	NO
13	2909	NO	YES	NO	NO	NO	NO	NO	NO	NO
14	2928	NO	YES	YES	NO	YES	NO	YES	YES	YES
15	2986	NO	YES	NO	NO	YES	NO	NO	NO	NO
16	3234	NO	NO	YES	NO	YES	NO	NO	NO	NO
17	3288	NO	YES	NO	NO	YES	NO	NO	NO	NO
18	3289	NO	YES	NO	NO	NO	NO	NO	NO	NO
19	3312	NO	YES	YES	YES	YES	YES	NO	NO	NO
20	3071	NO	YES	NO	NO	NO	NO	NO	NO	NO
21	3073	NO	NO	NO	NO	NO	NO	NO	NO	NO
22	2981	NO	NO	YES	NO	NO	NO	NO	NO	NO
23	130	NO	YES	YES	YES	NO	YES	NO	NO	NO
24	151	NO	NO	NO	NO	NO	NO	NO	NO	NO

Supporting Information Table S3: GenBank accession Number of sequences submitted in NCBI

Serial No.	Sequence ID	GenBank accession number
1	NICED/23-01/1914/hAdV	OR039269
2	NICED/23-02/1908/hAdV	OR089143
3	NICED/23-03/2280/hAdV	OR089144
4	NICED/23-04/2283/hAdV	OR089145
5	NICED/23-05/2220/hAdV	OR089146
6	NICED/23-06/2213/hAdV	OR089147
7	NICED/23-07/1995/hAdV	OR089148
8	NICED/23-08/1836/hAdV	OR089149
9	NICED/23-09/1639/hAdV	OR089150
10	NICED/23-10/2640/hAdV	OR130164
11	NICED/23-11/2788/hAdV	OR130165
12	NICED/23-12/2908/hAdV	OR130166
13	NICED/23-13/2909/hAdV	OR130167
14	NICED/23-14/2928/hAdV	OR130168
15	NICED/23-15/2986/hAdV	OR130169
16	NICED/23-16/3234/hAdV	OR130170
17	NICED/23-17/3288/hAdV	OR130171
18	NICED/23-18/3289/hAdV	OR130172
19	NICED/23-19/3312/hAdV	OR130173
20	NICED/22-20/3071/hAdV	OR130174
21	NICED/22-21/3073/hAdV	OR130175
22	NICED/22-22/2981/hAdV	OR130176
23	NICED/23-23/0130/hAdV	OR130177
24	NICED/23-24/0151/hAdV	OR130178
